# Cognitive Dysfunction in a Mouse Model of Cerebral Ischemia Influences Salivary Metabolomics

**DOI:** 10.3390/jcm10081698

**Published:** 2021-04-15

**Authors:** Masahiro To, Masahiro Sugimoto, Juri Saruta, Yuko Yamamoto, Wakako Sakaguchi, Akira Kawata, Masato Matsuo, Keiichi Tsukinoki

**Affiliations:** 1 Department of Oral Science, Graduate School of Dentistry, Kanagawa Dental University, Yokosuka 238-8580, Japan; m.tou@kdu.ac.jp (M.T.); mshrsgmt@gmail.com (M.S.); saruta@kdu.ac.jp (J.S.); sakaguchi@kdu.ac.jp (W.S.); kawata@kdu.ac.jp (A.K.); m.matsuo@kdu.ac.jp (M.M.); 2 Institute for Advanced Biosciences, Keio University, Tsuruoka 708-0813, Japan; 3 Health Promotion and Preemptive Medicine, Research and Development Center for Minimally Invasive Therapies, Tokyo Medical University, Tokyo 160-8402, Japan; 4 Department of Dental Hygiene, Junior College, Kanagawa Dental University, Yokosuka 238-8580, Japan; yamamoto.yuko@kdu.ac.jp

**Keywords:** dementia, cognitive impairment, cerebrovascular disease, saliva, metabolomics, BDNF

## Abstract

Vascular dementia, caused by cerebrovascular disease, is associated with cognitive impairment and reduced hippocampal metabolite levels. Specifically, cognitive impairment can be induced by decreased hippocampal brain-derived neurotrophic factor (BDNF) expression. The development of low or non-invasive biomarkers to characterize these diseases is an urgent task. Disturbance of metabolic pathways has been frequently observed in cognitive impairment, and salivary molecules also showed the potentials to reflect cognitive impairment. Therefore, we evaluated salivary metabolic profiles associated with altered hippocampal BDNF expression levels in a cerebral ischemia mouse model using metabolomic analyses. The effect of tacrine (a cholinesterase inhibitor) administration was also examined. The arteries of ICR mice were occluded with aneurysm clips to generate the cerebral ischemia model. Learning and memory performance was assessed using the elevated plus maze (EPM) test. Hippocampal and blood BDNF levels were quantified using an enzyme-linked immunosorbent assay. Glutamate decarboxylase 1 (*GAD1*) mRNA expression, is associated with cognitive impairment, was quantified by a real-time polymerase chain reaction. The EPM test revealed impaired spatial working memory in the cerebral ischemia mouse model; tacrine administration ameliorated this memory impairment. Cerebral ischemia suppressed *GAD1* expression by decreasing hippocampal BDNF expression. In total, seven salivary metabolites, such as trimethylamine *N*-oxide and putrescine, were changed by cognitive impairment and tacrine administration. Our data suggest that salivary metabolite patterns were associated with cognitive function.

## 1. Introduction

Dementia is a progressive neurodegenerative disease that causes cognitive impairment in older people. The number of people with dementia is estimated to be 35.6 million worldwide and is anticipated to double by 2030 [1]. Following Alzheimer’s disease, vascular dementia (VaD) is the second most common cause of dementia and is caused by cerebrovascular disease [2,3]. Risk factors include advanced age, underlying disease [4], and vasculature [5]. VaD can be further classified into subtypes, namely post-stroke dementia and multi-infarct dementia [4].

Multiple diagnostic criteria for VaD, as well as for its subtypes, are required to diagnose cognitive impairment [4]. This manifestation is induced by infarcts and atrophy in the brain, and coexistence with Alzheimer’s disease is frequent [3]. A VaD diagnosis is confirmed by brain neuroimaging (e.g., for white matter abnormalities) and cognitive assessments [4]. The pathogenic mechanism of VaD has been investigated in animal models [6], established using cerebral hypoperfusion [2,7]. This method has been widely used as a VaD model, with demonstrated behavioral deficits and neuronal damage in the hippocampus [8]. To further elucidate the pathobiology of VaD, many studies have used a cerebral ischemia model [6,9], which has provided evidence of the utility of neuroprotective agents in the brain [9]. Moreover, a previous study reported that brain-derived neurotrophic factor (BDNF) genetically modified mice had improved motor dysfunction following transient cerebral ischemia [10]. However, few studies have investigated diagnostic biomarkers for a cerebral ischemia animal model.

Animal models of dementia show impaired spatial learning and memory, suggesting hippocampal impairment [6,11,12,13]. Furthermore, in accordance with altered behavioral activity, hippocampal BDNF expression level is decreased [7,14]. BDNF plays an important role in learning and memory [15], and its polymorphism influences neurodegeneration in the hippocampus, as well as memory loss [16]. Brain dysfunction, including depression, can be reflected by hippocampal and blood levels of BDNF [17,18]. The link between BDNF and glutamate decarboxylase 1 (GAD1)–γ-aminobutyric acid (GABA) signaling in the hippocampus is critical for the onset of psychiatric disorders [19,20]. Furthermore, *GAD1* gene expression level shows an inverse relationship with age [21]. Cognitive decline is linked to reduced metabolite levels, including GABA, in the hippocampus [22]. A recent study indicated altered metabolites in the blood in an animal model of dementia [9]. The human study indicated that the biomarker in the blood is useful for cognitive impairment [23]. In Alzheimer’s disease, some salivary biomarkers in humans have been found using mass spectrometry or enzyme-linked immunosorbent assay (ELISA) [24,25,26].

It is known that antibacterial substances in saliva reflect changes in the brain more precisely than those in blood [27]. Additionally, the amount of drug administered in the brain is more precisely reflected in saliva than in blood [28]. Interestingly, saliva contains various metabolites produced from the _ENREF_1salivary gland, which provide a significant amount of information [29]. Saliva components have the potential to be used as biomarkers for several diseases [30], including dementia [31]; blood may also be used [32]. The measurement of salivary components can predict the onset of fatal disease [33]. In humans, the concentration of amyloid-β [32,34] and tau [35] in saliva reflected cognitive dysfunction. We have demonstrated that brain function, hippocampal BDNF, and GAD1-GABA signaling influence salivary BDNF levels in transgenic mice [36]. Furthermore, Ashton N.J. et al. suggested salivary biomarkers for neurodegenerative disorders [24]. To identify the biomarker for neurodegenerative disorders, various metabolites in blood and saliva have been investigated in experimental animals and humans [22,32,37]. However, the effects of cognitive impairment in a cerebral ischemia model on the salivary metabolic profile remain unknown.

Therefore, to elucidate whether cognitive impairment influences the salivary metabolic profile, we investigated the changes in salivary metabolites associated with altered hippocampal BDNF expression levels using a cerebral ischemia mouse model. Behavioral and salivary metabolome analyses were performed to determine cerebral ischemia biomarkers. Additionally, we evaluated the effect of the administration of tacrine (a cholinesterase inhibitor), which improves learning and memory deficits, on these alterations.

## 2. Experimental Section

### 2.1. Animals

Thirty-two eight-week-old male ICR mice (SLC Japan Inc., Shizuoka, Japan) were randomized to four groups (sham, ischemia, ischemia + saline, ischemia + tacrine) and were group-housed (4 per cage) with access to food and water ad libitum. Mice were maintained under pathogen-free conditions in a temperature-controlled room, with a light/dark cycle of 12 h (light off at 7:00 p.m.). All procedures were performed according to the guidelines for Animal Experiments of Kanagawa Dental University and were approved by the ethics committee for Animal Experiments of Kanagawa Dental University (15-032).

### 2.2. Experimental Design

The surgical operation for inducing cerebral ischemia was performed as described previously [13]. Mice were anesthetized with medetomidine hydrochloride, midazolam, and butorphanol tartrate, and the bilateral common carotid arteries were carefully separated from the cervical sympathetic and vagus nerves through a ventral cervical incision. The arteries were occluded with aneurysm clips for 20 min, and hypotension was produced by blood withdrawal (0.3 mL) from the tail during the ischemic operation. Animals that received the same surgical operation without carotid clamping and bleeding served as sham-operated controls (sham group).

In the ischemia + tacrine group, tacrine (9-amino-1,2,3,4-tetrahydro-acridine HCl; Sigma-Aldrich Co., St. Louis, MO, USA), a cholinesterase inhibitor, was administered intraperitoneally (2.5 mg/kg) according to a previous study [31]. The sham treatment group was injected with saline (ischemia + saline group). Tacrine/saline administration was performed daily for seven days, at around 3 p.m.

### 2.3. Behavioral Tests

The behavioral test of Itoh et al. [38] was conducted with several modifications. Mice from each group underwent the elevated plus maze (EPM) test. The testing instrument was surrounded by a black curtain and was illuminated with fluorescent light (light density was 23–25 lx). The EPM test consists of a plus-shaped maze with two open arms (30 × 5 cm) and two closed arms (30 × 5 × 20 cm) that spread out from a central platform (5 × 5 cm) and is elevated to a height of 50 cm from the floor. The mouse was placed at the end of an open arm, and the time taken for the mouse to move from the open arm to either of the closed arms (transfer latency) was recorded. If the mouse did not enter a closed arm within 60 s, the transfer latency was recorded as 60 s. All behavioral tests were conducted between 1 p.m. and 7 p.m., and the maze was cleaned with 30% ethanol solution after each trial. The test was repeated over five successive days [13,38].

### 2.4. ELISA

All samples were collected after 7 p.m. Hippocampal and blood BDNF levels were quantified using sandwich ELISA (ChemiKine™, MERCK MILLIPORE, Billerica, MA, USA) according to previous studies [39]. After HRP conjugation and color development, each sample was measured at a wavelength of 450/570 nm. The content was quantified using a standard curve generated using known amounts of BDNF, with a detection sensitivity of <15 pg/mL. BDNF level measurements were adjusted for the tissue wet weight.

### 2.5. Real-Time Polymerase Chain Reaction (PCR)

The instrumentation and conditions used for real-time PCR were as described previously [39]. RNA isolation from hippocampal tissue and cDNA synthesis were performed as previously described [39]. Real-time PCR was performed using the LightCycler system (Roche Diagnostics Ltd., West Sussex, UK) according to the manufacturer’s instructions. The primer sequences used to amplify *GAD1* were 5′-TCCTGGTTGACTGTAGAGACAC-3′ (forward) and 5′-CATATTGGTATTGGCAGTCGAT-3′ (reverse). Primers were designed and synthesized by Nihon Gene Research Laboratory (Sendai, Miyagi, Japan). The thermocycling parameters for *GAD1* were: 95 °C for 10 min, followed by 35 cycles at 95 °C for 10 s, 62 °C for 10 s, and 72 °C for 10 s. Mouse β-actin (*Actb*) was used as the housekeeping gene. Gene expression is indicated by the ratio of *GAD1* transcript number to *Actb* transcript number.

### 2.6. Metabolomic Analysis

#### 2.6.1. Saliva Sample Collection

After the behavioral test, each mouse was injected intraperitoneally with pilocarpine-HCl (Sanpilo 1%, 1 mg/kg; Santen Pharmaceutical Co. Ltd., Osaka, Japan) to induce salivary secretion [39]. We carefully collected saliva from the oral cavity using capillaries (Ringcaps; Hirschmann Laborgerate GmbH & Co. KG, Eberstadt, Germany). The collected sample (100–150 μL) was immediately placed on ice and stored at −80 °C until further use. The instrumentation and measurement conditions used for capillary electrophoresis–time-of-flight–mass spectrometry (CE-TOF-MS) were as previously described [40,41], with slight modifications.

#### 2.6.2. Saliva Metabolomics

Before the metabolome analyses, frozen saliva was thawed at 4 °C, and 100 μL of the saliva sample was centrifuged at 9100× *g* for 3 h at 4 °C, with a 5 kDa cut-off filter (Millipore, Billerica, MA, USA). The filtrate of the saliva sample (45 μL) was added to 5 μL of Milli-Q water containing internal standards and 20 mM each of methionine sulfone (Wako, Osaka, Japan), D-camphor-10-sulfonic acid (Wako), 2-(n-morpholino) ethanesulfonic acid (Dojindo, Kumamoto, Japan), 3-aminopyrrolidine (Sigma-Aldrich Japan, Tokyo, Japan), and trimesate (Wako). This mixture was centrifuged at 4550× *g* for 1 min at 4 °C, and 7 μL was transferred to a vial for CE-TOF-MS analysis. The measurement condition and instrument parameters for cation and anion profiles were as described elsewhere [42]. Raw data were analyzed using MasterHands [32,43] to produce a concentration matrix. The concentration of each metabolite was calculated using the ratio of the internal standards to the standard mixture.

### 2.7. Statistical Analyses

Statistical analyses were performed using GraphPad Prism (ver 8.4.3, GraphPad Software Inc., San Diego, CA, USA). Values are reported as the mean ± standard error of the mean. Experimental data were analyzed using a two-way (ischemia × trial) or one-way analysis of variance (ANOVA), followed by Tukey’s test. Partial least squares discriminant analysis (PLS-DA) and principal component analysis (PCA) was conducted using MetaboAnalyst (https://www.metaboanalyst.ca/, accessed on 23 February 2021) and a heatmap was created using MeV TM4 (http://mev.tm4.org/, accessed on 23 February 2021). The concentrations of the salivary metabolites among the four groups were assessed with one-way analysis of variance (ANOVA). *p*-values were adjusted by false discovery rate (Benjamini and Hochberg method) to yield q-values, considering multiple independent tests. Data were compared using the Mann–Whitney test. *p*-values < 0.05 were considered statistically significant. R-language (ver 4.0.2, https://www.R-project.org/, accessed on 23 February 2021) was used to create display items.

## 3. Results

### 3.1. Effects of Cerebral Ischemia on Learning and Memory Performance

**Figure 1:**
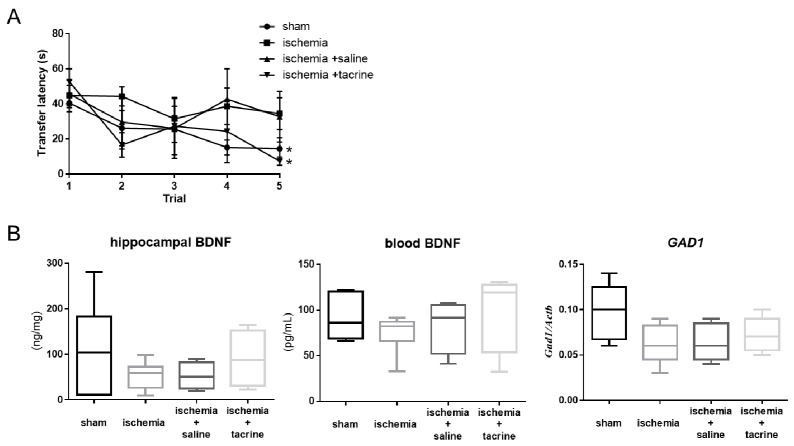
Behavioral performance and brain-derived neurotrophic factor (BDNF) and *GAD1* expression patterns in a cerebral ischemia mouse model. (**A**) The transfer latency in the elevated plus-maze test was significantly different between five trials in each group. Differences between each group were assessed using two-way ANOVA (each group: *n* = 4–6). * *p* <0.05. (**B**) Hippocampal BDNF and *GAD1* expression levels were lower in the ischemia group than in the sham group. Differences between each group were assessed using ANOVA (each group: *n* = 4–6).

To evaluate the effect of cerebral ischemia on learning and memory performance, we assessed the differences in transfer latency on the EPM test between the sham and ischemia groups. The transfer latency significantly differed between the first trial (40.5 ± 4.9 s) and the fifth trial (14.5 ± 6.3 s) in the sham group (*p* < 0.05; Figure 1A). However, in the ischemia group, there was no difference in the transfer latency between the first trial (44.8 ± 5.8 s) and the fifth trial (34.5 ± 9.1 s). Additionally, in the ischemia + tacrine group, the transfer latency was significantly lower at the fifth trial (7.3 ± 1.8 s) than at the first trial (52.7 ± 5.7 s, *p* < 0.05), which was similar to that in the sham group. These results suggest that spatial memory was impaired by cerebral ischemia and that tacrine administration ameliorated this deficit.

### 3.2. Cerebral Ischemia Affected BDNF-Associated Factors in the Hippocampus

To investigate the effect of cerebral ischemia on hippocampal and blood BDNF, BDNF levels were measured using ELISA. Hippocampal BDNF levels tended to be lower in the ischemia group (53.67 ± 12.43 ng/mg) than in the sham group (110.20 ± 41.44 ng/mg, *p* = 0.29; Figure 1B). Additionally, with tacrine administration, the BDNF level was equal to that in the sham group (ischemia + saline: 52.76 ± 15.03 ng/mg, ischemia + tacrine: 90.27 ± 31.52 ng/mg, *p* = 0.68). Blood BDNF levels also tended to be lower in the ischemia group (75.27 ± 8.66 pg/mL) than in the sham group (91.67 ± 10.57 pg/mL, *p* = 0.59), and this tendency was more pronounced with tacrine administration (ischemia + saline: 75.03 ± 17.27 pg/mL, ischemia + tacrine: 90.40 ± 29.07 pg/mL, *p* = 0.79; Figure 1B).

We investigated the impact of cerebral ischemia on the BDNF-related pathway in the hippocampus, focusing on *GAD1*, which is a GABA-associated factor. *GAD1* mRNA expression in the hippocampus was lower in the ischemia group (0.06 ± 0.01) than in the sham group (0.10 ± 0.01; Figure 1B). Additionally, to further clarify the effect of brain ischemia in the hippocampus, we analyzed *GAD1* mRNA levels in the hippocampus of tacrine-administered mice. *GAD1* expression tended to increase slightly more in the ischemia + tacrine group (0.07 ± 0.01) than in the ischemia + saline group (0.06 ± 0.01, *p* = 0.83). These results suggest that cerebral ischemia suppresses *GAD1* expression by decreasing BDNF levels in the hippocampus.

### 3.3. Effects of Cerebral Ischemia on Salivary Metabolite Profiles

**Figure 2:**
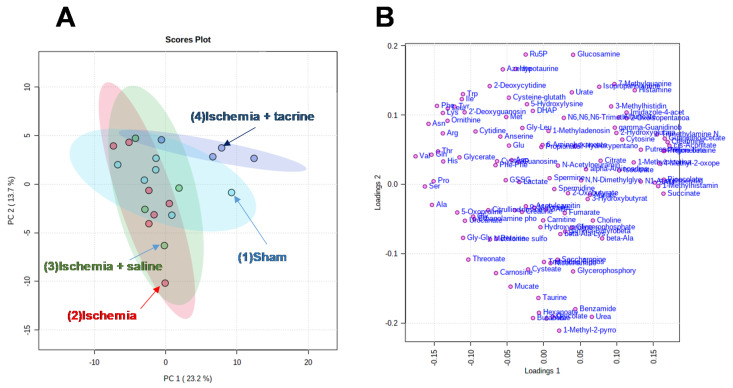
Principal component analysis of salivary metabolite concentrations. (**A**) Score plots and (**B**) loading plots are shown. Metabolite concentrations of each sample were normalized by the pooled sample from the sham group, after which a log transformation was carried out, and an auto-scaling option was used.

**Figure 3:**
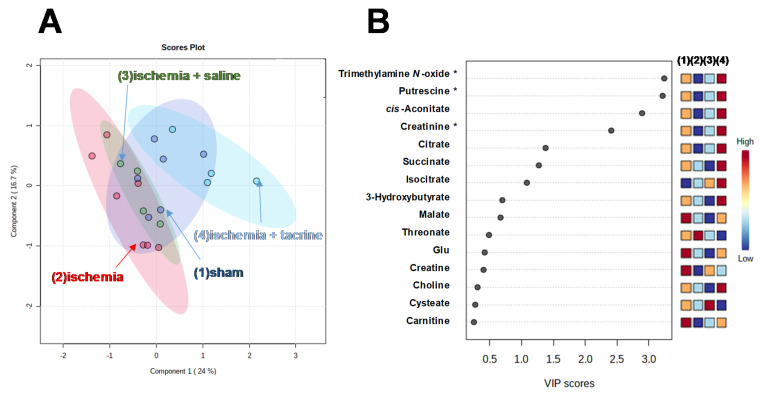
PLS-DA of salivary metabolite concentrations. (**A**) Score plots. (**B**) VIP scores. The metabolite detected in detection limits in more than 50% of the samples were selected and, of these, the metabolites showing variance value within the top 50% were analyzed. A total of 42 metabolites were used. The developed model yielded *R*^2^ = 0.72 and *Q*^2^ = 0.40 using two principal components. ANOVA tests were conducted for each metabolite, and *p*-values were adjusted by FDA. The metabolites showing *q*-value < 0.05 are shown with * in the VIP score. The right four column panels indicate the heatmap of four groups. The labels (1)–(4) on the heatmap correspond to the groups shown in (**A**), e.g., (1) sham group.

**Figure 4:**
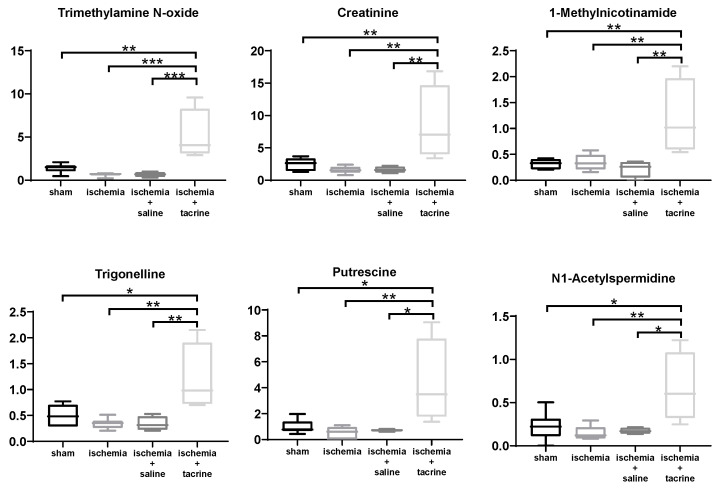
Changes in salivary metabolite concentrations due to brain ischemia (each group: *n* = 4–6). All six metabolites showed *q* < 0.05 (ANOVA test adjusted by FDR). The horizontal bars of each box and whiskers indicate 0, 25, 50, 75, 100% of the data. * *p* < 0.05, ** *p* < 0.01, *** *p* < 0.001 (Tukey’s multiple comparisons test as a post-test of ANOVA).

The metabolomic analyses identified and quantified 150 metabolites; of these, 114 metabolites detected in >50% of the samples were used in PCA analyses. The salivary metabolic profiles of all mice are presented in Figure 2, Figure 3 and Figure 4.

To compare the overall metabolic profiles of each mouse in all of the groups, a principal component analysis (PCA) was performed on detectable metabolites (Figure 2A). In the score plot, each point represents each mouse in all of the groups, with closer points indicating a greater similarity between salivary metabolite concentration patterns. Each point on the loading plot represents one saliva metabolite (Figure 2B). At the top left area of the score plots, both the first and second principal components (PC1 and PC2) were greater than 0, and samples of the four groups overlapped (Figure 2A). Most of the amino acids were observed at the corresponding area of the loading plots, indicating that the amino acids did not differ among the four groups (Figure 2B). Several samples in the ischemia group (red) were located in the bottom area (PC2 < 0), and all samples of the ischemia + tacrine group (dark blue) were located in the top area (PC2 > 0); additionally, several samples were located in the right area (PC1 > 0) of the score plots (Figure 2A). These two groups were the most different, with the other two groups (ischemia + saline, light green; sham, light blue) located between them.

To identify the metabolite contributing to the separation of the four groups, PLS-DA was also conducted (Figure 3A). There are partial overlaps among the four groups. However, the ischemia group was located at the left (PC1 < 0), whereas the ischemia + tacrine group was located at the opposite side (PC1 > 0). The other two groups were located between them. Metabolites with high variable importance in projection (VIP) score contribute to the separation of these groups. A total of seven metabolites showed relatively high VIP scores (VIP > 1.0) (Figure 3B). Especially, the top four metabolites showed high VIP > 2.0, whereas the rest showed VIP < 1.5. Among them, Trimethylamine *N*-oxide, putrescine, and *cis*-aconitate showed a significant level of ANOVA tests (*q*-value < 0.05). According to the heatmap (Figure 3B), the top five metabolites showed a similar concentration pattern, e.g., compared to the sham group (1), ischemia and ischemia + saline groups (2 and 3) showed lower concentrations, whereas the ischemia + tacrine group (4) showed a higher concentration.

Salivary metabolite concentrations were compared among the four groups, and 15 metabolites showed *p*-values < 0.05 using the ANOVA test. Considering alpha error caused by the multiple independent tests, false discovery rate (FDR) correction was used to yield *q*-values, and six metabolites showed *q*-values < 0.05. These metabolites included trimethylamine *N*-oxide (*p*-value = 0.0002, *q*-value = 0.017), creatinine (*p*-value = 0.0019, *q*-value = 0.042), 1-methylnicotinamide (*p*-value = 0.0019, *q*-value = 0.042), trigonelline (*p*-value = 0.02, *q*-value = 0.042), putrescine (*p*-value = 0.035, *q*-value = 0.050), and *N*^1^-acetylspermidine (*p*-value = 0.036, *q*-value = 0.050). The concentrations of these metabolites are depicted in Figure 4. As post-test, Tukey’s multiple comparisons were conducted. Among these six metabolites, ischemia+tacrine showed significant differences compared to the other groups.

## 4. Discussion

The current study demonstrated the effect of impaired cognitive function in a cerebral ischemia model on salivary components using a metabolomics approach. Recent metabolomic studies have reported that cognitive dysfunction affects various molecular factors in the hippocampus [21] and blood [11]. Furthermore, a metabolomic analysis has provided evidence for cancer-specific profiles in saliva [32]. Additionally, our previous study demonstrated the effects of physiological or environmental factors on salivary metabolomic profiles [29]. In the current study, a cerebrovascular disease mouse model with impaired spatial memory showed altered salivary metabolomic profiles. Moreover, salivary metabolites were restored to control levels after improvements in cognitive function. These results suggest that salivary components can reflect cognitive function.

In our mouse model, cerebral ischemia impaired cognitive function. Previous studies have reported on this mouse model, which is the most-used model of VaD [6,12]. This mouse model demonstrates deficits in spatial memory in the Morris water maze test [12,13,44]. Consistent with previous findings, the current study demonstrated cognitive function impairment in this mouse model on the EPM test. In this test, we found that the model mice had a significantly longer latency time than sham-operated mice. In the EPM test, the transfer latency is the parameter used to reflect learning and memory [38,45]. Moreover, transient cerebral ischemia induces neurodegeneration in the hippocampus by increasing oxidative stress and neuroinflammation [12,46]. Additionally, neuroprotective drugs improve brain disorders caused by transient cerebral ischemia [11]. Negren et al. investigated the effects of BDNF on brain dysfunction caused by transient cerebral ischemia [12]. Furthermore, inhibition of *GAD* expression is associated with cognitive decline [47]. Our study showed that the levels of BDNF and *GAD1* expression were significantly reduced in the model mice. In patients and animal models with cerebrovascular disease, the BDNF levels are reduced in the hippocampus and blood, resulting in cognition decline [7,48]. Moreover, increased hippocampal BDNF levels, as a result of peripheral administration, enables mice to recover from depression [17]. Similar to a previous study, our model mice showed decreased blood BDNF levels due to cerebral ischemia. Additionally, alterations in the BDNF and *GAD1* levels affect behavioral actions mediated by GABA signaling [36]. Our results also showed that the administration of tacrine, a cholinesterase inhibitor, restored the BDNF levels and behavioral activity, even though it did not restore the decrease in *GAD1* expression. A recent study showed that tacrine at a higher dose than that in the current study inhibits *GAD1* expression [49]. Additionally, previous studies have shown that the administration of tacrine improves brain dysfunction, consistent with our results [50,51]. Our study showed that this mouse model experienced impaired cognitive function as a result of transient cerebral ischemia and that this dysfunction was restored by administrating tacrine.

The current study also showed that the salivary metabolic profile was affected by cerebral ischemia in a cerebral ischemia mouse model. Li et al. [11] conducted a metabolomics study of blood and reported changes in various metabolites associated with cognitive deficits. Their study showed an increase in multiple metabolites and a decrease in phenylalanine associated with cognitive dysfunction. Moreover, the levels of these metabolites were increased to control levels by improving cognitive function. Our findings demonstrated that the multivariate analyses using PLS-DA revealed seven metabolites showed relatively high VIP > 1.0, e.g., these metabolites contribute to the separation of the four groups. Among seven metabolites, putrescine is one polyamine that is synthesized from ornithine by ornithine decarboxylase, and putrescine is metabolized into spermidine by spermidine synthase and into spermine by spermine synthase. *N*^1^-Acetylspermidine is also an acetylated form of polyamine. The relationship between these polyamines and brain functions, for example the association between spermidine intake and hippocampal volume in older subjects, has been reported [52]. Spermidine has functions that include a protective effect against traumatic brain injury [53]. Serum metabolomics found significantly lower putrescine concentration in Alzheimer’s disease compared to mild cognitive impairment patients [54]. Creatinine is a breakdown product of creatine phosphate in muscle and increasing creatinine concentration in serum of Alzheimer’s disease was observed [55]. The change of this metabolite in the saliva of neurodegenerative dementia patients was also reported even though the number of subjects was small [56]. 1-Methylnicotinamide is one of the intermediate metabolites in nicotinamide metabolism. This metabolite attenuated lipopolysaccharide (LPS)-induced cognitive deficits via targeting neuroinflammation and neuronal apoptosis [57]. Trimethylamine *N*-oxide is an oxidation product of trimethylamine, and its change in plasma and urine has been associated with various diseases, such as schizophrenia [58]. Taken together, the salivary metabolites in our results can be considered biomarkers of brain dysfunction.

Our study has several limitations. First, the sample size in our study was small. In our study, 32 mice (*n* = 8 in each group) were used for analyses. However, since some mice showed abnormal reactions (long resting-state time, etc.), or died after the operation or during sampling, they were excluded from the analysis of salivary metabolites. All of the other mice showed some individual differences in behavioral activity, expression of each evaluated molecule, and metabolites. Future studies with several animals are needed to validate our results. Second, this study showed only salivary metabolic profiles without analyzing blood samples. The effect of cognitive function on systemic metabolism should be analyzed, and the injection of isotope-labeled metabolites and their trace could also be used to understand the transfer of the metabolites among various matrices. Evaluation of the change of salivary molecules associated with neurodegenerative disorders has frequently been reported [24,25]. Several clinical studies revealed that salivary metabolites, such as amino acids and peptides, showed significant differences among the subjects with Alzheimer’s disease (AD) and mild cognitive impairment as well as healthy controls, though there is still room to validate their sensitivity and specificity via a large cohort study [24].

This study focused on salivary metabolites since their non-invasive collection would provide benefit as a clinical test. Non-metabolite biomarkers, such as C-reactive proteins and tumor necrosis factors in saliva, were elevated in the subjects with AD or coronary heart diseases compared to healthy controls, which indicates that these markers showed no specificity to AD [33]. However, the mechanisms underlying the effect of ischemia on BDNF signaling remain unclear in our study, and thus, a comprehensive analysis of metabolites is necessary. A previous report indicated that tyrosine kinase B, a BDNF receptor, was decreased in the hippocampus of individuals with psychiatric disorders [59]. The hippocampal BDNF/cAMP-responsive element-binding protein signaling pathway has also been related to cognitive function [60]. Thus, future studies should investigate other salivary biomarkers and the expression of tyrosine kinase B and cAMP-responsive element-binding protein, alongside its relation to BDNF polymorphisms, which influence neurodegeneration in the hippocampus, and salivary metabolites. Thirdly, salivary metabolites may vary depending on the quality and diurnal variation of saliva samples. All samples were collected at the same time of the day in our study. Thus, future studies should validate these findings at different times of the day. Additionally, due to the limited amount of saliva and hippocampal tissue that can be collected from mice, the concentration of salivary BDNF and hippocampal metabolomics could not be measured. Future studies should evaluate these data in this model.

## 5. Conclusions

In summary, the analysis of salivary metabolic profiles in cerebrovascular disease model mice showed that metabolite levels were altered by cerebral ischemia; furthermore, the concentrations of some metabolites were altered by tacrine administration. Our data suggest that salivary metabolite patterns were associated with cognitive function.

## Data Availability

The datasets generated and analyzed during the current study are available from the corresponding author on reasonable request.

## References

[1] Wortmann M. (2012). Dementia: A global health priority-highlights from an ADI and World Health Organization report. Alzheimers Res. Ther..

[2] Wang Z., Fan J., Wang J., Li Y., Duan D., Du G., Wang Q. (2016). Chronic cerebral hypoperfusion induces long-lasting cognitive deficits accompanied by long-term hippocampal silent synapses increase in rats. Behav. Brain Res..

[3] Iadecola C. (2013). The Pathobiology of Vascular Dementia. Neuron.

[4] Moorhouse P., Rockwood K. (2008). Vascular cognitive impairment: Current concepts and clinical developments. Lancet Neurol..

[5] Wallin A., Sjögren M., Edman Å., Blennow K., Regland B. (2000). Symptoms, vascular risk factors and blood-brain barrier function in relation to CT white-matter changes in dementia. Eur. Neurol..

[6] Jiwa N.S., Garrard P., Hainsworth A.H. (2010). Experimental models of vascular dementia and vascular cognitive impairment: A systematic review. J. Neurochem..

[7] Zhang N., Xing M., Wang Y., Tao H., Cheng Y. (2015). Repetitive transcranial magnetic stimulation enhances spatial learning and synaptic plasticity via the VEGF and BDNF–NMDAR pathways in a rat model of vascular dementia. Neuroscience.

[8] Farkas E., Luiten P.G., Bari F. (2007). Permanent, bilateral common carotid artery occlusion in the rat: A model for chronic cerebral hypoperfusion-related neurodegenerative diseases. Brain Res. Rev..

[9] Mori M.A., Meyer E., da Silva F.F., Milani H., Guimarães F.S., de Oliveira R.M.W. (2021). Differential contribution of CB1, CB2, 5-HT1A, and PPAR-γ receptors to cannabidiol effects on ischemia-induced emotional and cognitive impairments. Eur. J. Neurosci..

[10] Nygren J., Kokaia M., Wieloch T. (2006). Decreased expression of brain-derived neurotrophic factor in BDNF+/− mice is associated with enhanced recovery of motor performance and increased neuroblast number following experimental stroke. J. Neurosci. Res..

[11] Li J., Liu Y., Li W., Wang Z., Guo P., Li L., Li N. (2018). Metabolic profiling of the effects of ginsenoside Re in an Alzheimer’s disease mouse model. Behav. Brain Res..

[12] Truiti M.T., Soares L., Longhini R., Milani H., Nakamura C.V., Mello J.C.P., De Oliveira R.M.W. (2015). Trichilia catigua ethyl-acetate fraction protects against cognitive impairments and hippocampal cell death induced by bilateral common carotid occlusion in mice. J. Ethnopharmacol..

[13] Zhao Q., Murakami Y., Tohda M., Watanabe H., Matsumoto K. (2005). Preventive Effect of Chotosan, a Kampo Medicine, on Transient Ischemia-Induced Learning Deficit Is Mediated by Stimulation of Muscarinic M1 But Not Nicotinic Receptor. Biol. Pharm. Bull..

[14] Afshar S., Shahidi S., Rohani A.H., Komaki A., Asl S.S. (2018). The effect of NAD-299 and TCB-2 on learning and memory, hippocampal BDNF levels and amyloid plaques in Streptozotocin-induced memory deficits in male rats. Psychopharmacology.

[15] Monteggia L.M., Barrot M., Powell C.M., Berton O., Galanis V., Gemelli T., Meuth S., Nagy A., Greene R.W., Nestler E.J. (2004). Essential role of brain-derived neurotrophic factor in adult hippocampal function. Proc. Natl. Acad. Sci. USA.

[16] Lim Y.Y., Rainey-Smith S., Lim Y., Laws S.M., Gupta V., Porter T., Bourgeat P., Ames D., Fowler C., Salvado O. (2017). BDNF Val66Met in preclinical Alzheimer’s disease is associated with short-term changes in episodic memory and hippocampal volume but not serum mBDNF. Int. Psychogeriatr..

[17] Schmidt H.D., Duman R.S. (2010). Peripheral BDNF Produces Antidepressant-Like Effects in Cellular and Behavioral Models. Neuropsychopharmacology.

[18] Gil-Ad I., Portnoy M., Tarasenko I., Bidder M., Kramer M., Taler M., Weizman A. (2014). A novel analog of olanzapine linked to sarcosinyl moiety (PGW5) demonstrates high efficacy and good safety profile in mouse models of schizophrenia. Eur. Neuropsychopharmacol..

[19] Makinson R., Lundgren K.H., Seroogy K.B., Herman J.P. (2015). Chronic social subordination stress modulates glutamic acid decarboxylase (GAD) 67 mRNA expression in central stress circuits. Physiol. Behav..

[20] Subburaju S., Benes F.M. (2012). Induction of the GABA Cell Phenotype: An In Vitro Model for Studying Neurodevelopmental Disorders. PLoS ONE.

[21] Siegmund K.D., Connor C.M., Campan M., Long T.I., Weisenberger D.J., Biniszkiewicz D., Jaenisch R., Laird P.W., Akbarian S. (2007). DNA Methylation in the Human Cerebral Cortex Is Dynamically Regulated throughout the Life Span and Involves Differentiated Neurons. PLoS ONE.

[22] Zheng Y., Yang Y., Dong B., Zheng H., Lin X., Du Y., Li X., Zhao L., Gao H. (2016). Metabonomic profiles delineate potential role of glutamate-glutamine cycle in db/db mice with diabetes-associated cognitive decline. Mol. Brain.

[23] Wang Z., Wang R., Li Y., Li M., Zhang Y., Jiang L., Fan J., Wang Q., Yang D. (2021). Plasma Neurofilament Light Chain as a Predictive Biomarker for Post-stroke Cognitive Impairment: A Prospective Cohort Study. Front. Aging Neurosci..

[24] Bouftas M. (2020). A Systematic Review on the Feasibility of Salivary Biomarkers for Alzheimer’s Disease. J. Prev. Alzheimers Dis..

[25] Ashton N.J., Ide M., Zetterberg H., Blennow K. (2019). Salivary Biomarkers for Alzheimer’s Disease and Related Disorders. Neurol. Ther..

[26] Lee M., Guo J.-P., Kennedy K., McGeer E.G., McGeer P.L. (2016). A Method for Diagnosing Alzheimer’s Disease Based on Salivary Amyloid-β Protein 42 Levels. J. Alzheimer’s Dis..

[27] Hayashi T., To M., Saruta J., Sato C., Yamamoto Y., Kondo Y., Shimizu T., Kamata Y., Tsukinoki K. (2017). Salivary lactoferrin is transferred into the brain via the sublingual route. Biosci. Biotechnol. Biochem..

[28] Takai N., Eto K., Uchihashi K., Yamaguchi M., Nishikawa Y. (2006). Correlation of haloperidol levels between submandibular saliva and brain in the rat. Arch. Oral Biol..

[29] Sugimoto M., Saruta J., Matsuki C., To M., Onuma H., Kaneko M., Soga T., Tomita M., Tsukinoki K. (2012). Physiological and environmental parameters associated with mass spectrometry-based salivary metabolomic profiles. Metabolomics.

[30] Sugimoto M. (2020). Salivary metabolomics for cancer detection. Expert Rev. Proteom..

[31] François M., Bull C.F., Fenech M.F., Leifert W.R. (2018). Current State of Saliva Biomarkers for Aging and Alzheimer’s Disease. Curr. Alzheimer Res..

[32] Sugimoto M., Wong D.T., Hirayama A., Soga T., Tomita M. (2009). Capillary electrophoresis mass spectrometry-based saliva metabolomics identified oral, breast and pancreatic cancer-specific profiles. Metabolomics.

[33] McGeer P.L., Lee M., Kennedy K., McGeer E.G., Geer M. (2020). Saliva Diagnosis as a Disease Predictor. J. Clin. Med..

[34] Sabbagh M.N., Shi J., Lee M., Arnold L., Al-Hasan Y., Heim J., McGeer P. (2018). Salivary beta amyloid protein levels are detectable and differentiate patients with Alzheimer’s disease dementia from normal controls: Preliminary findings. BMC Neurol..

[35] Shi M., Sui Y.-T., Peskind E.R., Li G., Hwang H., Devic I., Ginghina C., Edgar J.S., Pan C., Goodlett D.R. (2011). Salivary Tau Species are Potential Biomarkers of Alzheimer’s Disease. J. Alzheimers Dis..

[36] Saruta J., To M., Sugimoto M., Yamamoto Y., Shimizu T., Nakagawa Y., Inoue H., Saito I., Tsukinoki K. (2017). Salivary Gland Derived BDNF Overexpression in Mice Exerts an Anxiolytic Effect. Int. J. Mol. Sci..

[37] Ralbovsky N.M., Halámková L., Wall K., Anderson-Hanley C., Lednev I.K. (2019). Screening for Alzheimer’s Disease Using Saliva: A New Approach Based on Machine Learning and Raman Hyperspectroscopy. J. Alzheimers Dis..

[38] Itoh J., Nabeshima T., Kameyama T. (1991). Utility of an elevated plus-maze for dissociation of amnesic and behavioral effects of drugs in mice. Eur. J. Pharmacol..

[39] Saruta J., Lee T., Shirasu M., Takahashi T., Sato C., Sato S., Tsukinoki K. (2010). Chronic stress affects the expression of brain-derived neurotrophic factor in rat salivary glands. Stress.

[40] Sugimoto M., Sakagami H., Yokote Y., Onuma H., Kaneko M., Mori M., Sakaguchi Y., Soga T., Tomita M. (2011). Non-targeted metabolite profiling in activated macrophage secretion. Metabolomics.

[41] Sugimoto M., Ota S., Kaneko M., Enomoto A., Soga T. (2020). Quantification of Salivary Charged Metabolites using Capillary Electrophoresis Time-of-flight-mass Spectrometry. Bio-Protocol.

[42] Ishikawa S., Sugimoto M., Kitabatake K., Sugano A., Nakamura M., Kaneko M., Ota S., Hiwatari K., Enomoto A., Soga T. (2016). Identification of salivary metabolomic biomarkers for oral cancer screening. Sci. Rep..

[43] Sugimoto M., Kawakami M., Robert M., Soga T., Tomita M. (2012). Bioinformatics Tools for Mass Spectroscopy-Based Metabolomic Data Processing and Analysis. Curr. Bioinform..

[44] Watanabe H., Zhao Q., Matsumoto K., Tohda M., Murakami Y., Zhang S.-H., Kang T.-H., Mahakunakorn P., Maruyama Y., Sakakibara I. (2003). Pharmacological evidence for antidementia effect of Choto-san (Gouteng-san), a traditional Kampo medicine. Pharmacol. Biochem. Behav..

[45] Itoh J., Nabeshima T., Kameyama T. (1990). Utility of an elevated plus-maze for the evaluation of memory in mice: Effects of nootropics, scopolamine and electroconvulsive shock. Psychopharmacology.

[46] Godinho J., De Oliveira R.M.W., De Sa-Nakanishi A.B., Bacarin C.C., Huzita C.H., Longhini R., Mello J.C.P., Nakamura C.V., Previdelli I.S., Ribeiro M.H.D.M. (2018). Ethyl-acetate fraction of Trichilia catigua restores long-term retrograde memory and reduces oxidative stress and inflammation after global cerebral ischemia in rats. Behav. Brain Res..

[47] Takagi M., Ishigaki Y., Uno K., Sawada S., Imai J., Kaneko K., Hasegawa Y., Yamada T., Tokita A., Iseki K. (2013). Cognitive dysfunction associated with anti-glutamic acid decarboxylase autoimmunity: A case-control study. BMC Neurol..

[48] Passaro A., Nora E.D., Morieri M.L., Soavi C., Sanz J.M., Zurlo A., Fellin R., Zuliani G. (2014). Brain-Derived Neurotrophic Factor Plasma Levels: Relationship with Dementia and Diabetes in the Elderly Population. J. Gerontol. Ser. A Boil. Sci. Med. Sci..

[49] Kumari E., Li K., Yang Z., Zhang T. (2020). Tacrine accelerates spatial long-term memory via improving impaired neural oscillations and modulating GAD isomers including neuro-receptors in the hippocampus of APP/PS1 AD mice. Brain Res. Bull..

[50] Zhao Q., Murakami Y., Tohda M., Obi R., Shimada Y., Matsumoto K. (2007). Chotosan, a Kampo Formula, Ameliorates Chronic Cerebral Hypoperfusion-Induced Deficits in Object Recognition Behaviors and Central Cholinergic Systems in Mice. J. Pharmacol. Sci..

[51] Murakami Y., Zhao Q., Harada K., Tohda M., Watanabe H., Matsumoto K. (2005). Choto-san, a Kampo formula, improves chronic cerebral hypoperfusion-induced spatial learning deficit via stimulation of muscarinic M receptor. Pharmacol. Biochem. Behav..

[52] Schwarz C., Horn N., Benson G., Calzado I.W., Wurdack K., Pechlaner R., Grittner U., Wirth M., Flöel A. (2020). Spermidine intake is associated with cortical thickness and hippocampal volume in older adults. NeuroImage.

[53] Huang J., Zhang H., Zhang J., Yu H., Lin Z., Cai Y. (2020). Spermidine Exhibits Protective Effects Against Traumatic Brain Injury. Cell. Mol. Neurobiol..

[54] Weng W.-C., Huang W.-Y., Tang H.-Y., Cheng M.-L., Chen K.-H. (2019). The Differences of Serum Metabolites Between Patients with Early-Stage Alzheimer’s Disease and Mild Cognitive Impairment. Front. Neurol..

[55] González-Domínguez R., García A., García-Barrera T., Barbas C., Gómez-Ariza J.L. (2014). Metabolomic profiling of serum in the progression of Alzheimer’s disease by capillary electrophoresis-mass spectrometry. Electrophoresis.

[56] Tsuruoka M., Hara J., Hirayama A., Sugimoto M., Soga T., Shankle W.R., Tomita M. (2013). Capillary electrophoresis-mass spectrometry-based metabolome analysis of serum and saliva from neurodegenerative dementia patients. Electrophoresis.

[57] Mu R.-H., Tan Y.-Z., Fu L.-L., Islam M.N., Hu M., Hong H., Tang S.-S. (2019). 1-Methylnicotinamide attenuates lipopolysaccharide-induced cognitive deficits via targeting neuroinflammation and neuronal apoptosis. Int. Immunopharmacol..

[58] Schulman A.N., Dienstag J.L., Jackson D.R., Hoofnagle J.H., Gerety R.J., Purcell R.H., Barker L.F. (1976). Hepatitis a Antigen Particles in Liver, Bile, and Stool of Chimpanzees. J. Infect. Dis..

[59] Ray M.T. (2011). Decreased BDNF, trkB-TK+ and GAD67 mRNA expression in the hippocampus of individuals with schizophrenia and mood disorders. J. Psychiatry Neurosci..

[60] Nakagawasai O., Lin J.-R., Odaira T., Takahashi K., Nemoto W., Moriguchi S., Yabuki Y., Kobayakawa Y., Fukunaga K., Nakada M. (2020). Scabronine G Methyl Ester Improves Memory-Related Behavior and Enhances Hippocampal Cell Proliferation and Long-Term Potentiation via the BDNF-CREB Pathway in Olfactory Bulbectomized Mice. Front. Pharmacol..

